# Using implementation facilitation to implement primary care mental health integration via clinical video telehealth in rural clinics: protocol for a hybrid type 2 cluster randomized stepped-wedge design

**DOI:** 10.1186/s13012-019-0875-5

**Published:** 2019-03-21

**Authors:** Richard R. Owen, Eva N. Woodward, Karen L. Drummond, Tisha L. Deen, Karen Anderson Oliver, Nancy J. Petersen, Scott S. Meit, John C. Fortney, JoAnn E. Kirchner

**Affiliations:** 1 VA Center for Mental Healthcare and Outcomes Research, Central Arkansas Veterans Healthcare System, 2200 Fort Roots Drive, North Little Rock, AR USA; 2 Department of Psychiatry, University of Arkansas for Medical Sciences, 4301 West Markham, Little Rock, AR USA; 3 Center for Implementation Research, University of Arkansas for Medical Sciences, 4301 West Markham, Little Rock, AR USA; 4 Central Arkansas Veterans Healthcare System, 2200 Fort Roots Drive, North Little Rock, AR USA; 5 William S. Middleton Memorial Veterans Hospital, 2500 Overlook Terrace, Madison, WI USA; 6 VA HSR&D Center for Innovations in Quality Effectiveness and Safety, Michael E. DeBakey VA Medical Center, Houston, TX USA; 7 Department of Medicine, Baylor College of Medicine, Houston, TX USA; 8 Health Services Research and Development, Center of Innovation for Veteran-Centered and Value-Driven Care, VA Puget Sound Health Care System, 1660 S. Columbian Way, Seattle, WA S-152 USA; 9 Department of Psychiatry and Behavioral Sciences, University of Washington, 1959 NE Pacific Street, Seattle, WA USA; 10 Department of Veterans Affairs, VA Quality Enhancement Research Initiative (QUERI) Program for Team-Based Behavioral Health, 2200 Fort Roots Drive, North Little Rock, AR USA

**Keywords:** Facilitation, Implementation, Primary care mental health integration, Integrated primary care, Rural veterans, Stepped-wedge design, Hybrid design

## Abstract

**Background:**

Integrating mental health providers into primary care clinics improves access to and outcomes of mental health care. In the Veterans Health Administration (VA) Primary Care Mental Health Integration (PCMHI) program, mental health providers are co-located in primary care clinics, but the implementation of this model is challenging outside large VA medical centers, especially for rural clinics without full mental health staffing. Long wait times for mental health care, little collaboration between mental health and primary care providers, and sub-optimal outcomes for rural veterans could result. Telehealth could be used to provide PCMHI to rural clinics; however, the clinical effectiveness of the tele-PCMHI model has not been tested. Based on evidence that implementation facilitation is an effective implementation strategy to increase uptake of PCMHI when delivered on-site at larger VA clinics, it is hypothesized that this strategy may also be effective with regard to ensuring adequate uptake of the tele-PCMHI model at rural VA clinics.

**Methods:**

This study is a hybrid type 2 pragmatic effectiveness-implementation trial of tele-PCMHI in six sites over 24 months. Tele-PCMHI, which will be delivered by clinical staff available in routine care settings, will be compared to usual care. Fidelity to the care model will be monitored but not controlled. We will use the Reach Effectiveness Adoption Implementation Maintenance (RE-AIM) framework to evaluate the patient-level clinical effectiveness of tele-PCMHI in rural VA clinics and also to evaluate the fidelity to and outcomes of the implementation strategy, implementation facilitation. The proposed study will employ a stepped-wedge design in which study sites sequentially begin implementation in three steps at 6-month intervals. Each step will include (1) a 6-month period of implementation planning, followed by (2) a 6-month period of active implementation, and (3) a final period of stepped-down implementation facilitation.

**Discussion:**

This study will evaluate the effectiveness of PCMHI in a novel setting and via a novel method (clinical video telehealth). We will test the feasibility of using implementation facilitation as an implementation strategy to deploy tele-PCMHI in rural VA clinics.

**Trial registration:**

ClinicalTrials.gov registration number NCT02713217. Registered on 18 March 2016

**Electronic supplementary material:**

The online version of this article (10.1186/s13012-019-0875-5) contains supplementary material, which is available to authorized users.

## Background

Integrating mental health care into primary care settings is a major priority in the Veterans Health Administration (VA) as well as in other health care settings [1, 2]. Within VA, Primary Care Mental Health Integration (PCMHI) increases access to mental and behavioral health care [3, 4], sustains patients in care [5], and improves health outcomes for patients with a wide variety of diagnoses, including depression, schizophrenia, and anxiety disorders [6–8]. PCMHI also reduces the use of more costly specialty mental health care services through treating appropriate behavioral health problems in the primary care setting [8]. However, PCMHI is complex because it requires changes to the structure of primary care clinics, funding allocation, leadership engagement, and patient flow, and may require additional training for staff [9].

Within VA, models for integrating mental health care into primary care settings include co-location of mental health providers in primary care clinics and care management models [10]. PCMHI was designed to be staffed by on-site psychotherapists (e.g., social workers, psychologists), consulting prescribers (e.g., psychiatrists, clinical pharmacists), and nurse care managers. The on-site mental health providers staff an open-access clinic facilitated by warm handoffs from primary care providers to mental health providers, preferably with the patient present. Patients (regardless of diagnoses) referred to the co-located mental health providers receive focused assessment and if needed, brief treatment. Those needing longer-term or more intensive treatment are referred to specialty mental health providers or clinics. Primary care providers can also choose to refer the patient (regardless of diagnosis) to a remote care manager directly, bypassing the co-located mental health providers (e.g., to monitor antidepressant adherence). Thus, PCMHI addresses a wide array of disorders and behavioral problems commonly seen in primary care settings.

Current mandates within the VA require co-located PCMHI services at VA Medical Centers and larger community-based outpatient clinics (CBOCs) [11]. Based on their experience in facilitating integrated care models in CBOC settings, our PCMHI and regional mental health operations partners report that, although PCMHI is desirable, implementation is extremely challenging for CBOCs that lack a full range of mental health staff. This often results in long wait times for mental health care, little collaboration between mental health providers and primary care providers, and could result in sub-optimal outcomes for rural veterans. In addition, the volume of veterans who need PCMHI care in these rural clinics does not justify the placement of a full-time PCMHI provider in the clinic. While some clinics have addressed this by having PCMHI staff available on an intermittent schedule, the intermittent availability of the PCMHI provider may or may not coincide with the clinic visits of the veterans needing these services.

### The innovation: tele-PCMHI

The innovation under study is PCMHI delivered via clinical video telehealth (“telehealth”), i.e., virtually co-locating mental health providers in CBOCs that lack on-site mental health care staff in primary care or lack sufficient capacity to meet the clinical need for PCMHI. A version of care management that was specifically tailored for such small CBOCs resulted in significant improvements in adherence to medication, treatment response, and remission of depression for patients assigned to the care management condition as compared with those in usual care [12]. In contrast, the clinical effectiveness of the co-located PCMHI model has not been tested in rural CBOCs. This project fills critical gaps in the scientific evidence base about the effectiveness of the innovation in this setting and how best to integrate the innovation in rural clinics by creating, adapting, and testing a tele mental health model of PCMHI (tele-PCMHI) for rural settings. If successful, tele-PCMHI will allow integrated mental health services to be implemented in areas where staffing co-located specialists is not feasible or cost-effective, including more primary care clinics serving rural patients.

### Implementation strategy: Implementation facilitation (IF)

Successful implementation is defined as the achievement of agreed goals, the uptake and institutionalization of the innovation, engaged stakeholders who “own” the innovation, and the minimization of variation related to context across implementation settings [13]. We will use the Integrated Promoting Action on Research Implementation in Health Services (i-PARIHS) framework to guide implementation strategies in this study. The i-PARIHS framework proposes that successful implementation (SI) of innovations is the result of the facilitation (Fac^n^) of an innovation (*I*) with recipients (*R*) in the inner and outer context (*C*) [SI = Fac^n^(*I* + *R* + *C*)] [13]. Among the i-PARIHS constructs, implementation facilitation (IF) is viewed as the essential ingredient, with designated facilitators activating implementation by assessing and guiding the recipients (both patients and providers) of tele-PCMHI through their contexts (rural primary care clinics). Facilitators may be internal or external to the implementation setting. For this study, we will use an IF team comprised of two facilitators—one facilitator internal to the region in which study sites are located and one facilitator external to the region. Both facilitators are trained in IF and one is an expert in the delivery of co-located PCMHI.

IF involves an integrated set of implementation strategies to promote adoption of tele-PCMHI. In this study, we will implement the tele-PCMHI model (the innovation) by facilitating the development of implementation plans by the clinical stakeholders (the recipients), guiding them about the application of the evidence base, and measuring fidelity to the core components of the innovation. In addition to providing expertise, study facilitators will also support problem-solving and provide ongoing technical support for developing data collection/analysis tools, informatics, and training materials. Because IF also emphasizes continuously revising an adapted innovation based on feedback during the implementation process to fit the site’s context, we expect the resulting tele-PCMHI model to be aligned with the policies and priorities of our operational partners, including the national VA offices for mental health and primary care services. In addition, this work should lead to an adapted innovation that is robust, user-friendly, and feasible to deploy in real-world practice settings.

### Aims and objectives of the current project

The goals of this study are to (1) generate the scientific evidence needed to justify the national dissemination of the tele-PCMHI model adapted to accommodate the clinical context of rural CBOCs and (2) test the feasibility of using IF to deploy the tele-PCMHI model in rural VA CBOCs that lack on-site capacity to provide PCMHI. Tele-PCMHI will be compared to usual care in a pragmatic trial, in which the innovation will be delivered by clinical staff available in routine care settings and fidelity will be monitored but not controlled. Because this hybrid type 2 trial involves *both* implementation and effectiveness activities and evaluations, there is an implementation aim and an effectiveness aim.

#### Specific aim 1 (implementation aim)

Using an expert panel, identify the core components of the tele-PCMHI model for CBOCs and implement this model in six CBOCs, using a facilitated Plan-Do-Study-Act (PDSA) process, working with key clinical staff at sites to adapt the model so that the PCMHI providers deliver evidence-based “co-located” collaborative care using telehealth. This aim involves typical quality improvement activities rather than research.

#### Specific aim 2 (effectiveness aim)

Conduct a hybrid type 2 [14] pragmatic effectiveness-implementation trial of tele-PCMHI by assessing outcomes including provider reach into the patient population, effectiveness at improving clinical outcomes, adoption by providers, and implementation and innovation fidelity. This evaluation assesses the effectiveness of the implementation strategy as well as patient outcomes of the tele-PCMHI model.

If the proposed study is successful, our clinical partners in VA leadership will use the results to justify implementation of the tele-PCMHI model in CBOCs, including those that serve rural-dwelling veterans, that have not yet implemented PCMHI.

## Methods

### Site selection

This study will be conducted in conjunction with two parent VA Medical Centers within a single region or Veterans Integrated Services Network (VISN) in the southern USA. Each parent VA Medical Center will identify CBOCs that lack on-site full-time psychiatrists and psychologists or report a major unmet need for PCMHI services. Final selection will be based on recommendations from VISN mental health leadership and VA Medical Center leaders’ willingness to participate.

All sites will have on-site licensed clinical social workers (LCSWs), mental health telephone care managers, and interactive video equipment available. The study will provide a tele-psychologist to receive warm handoffs and provide open access to PCMHI services. During implementation planning (see below), we will work with mental health leaders at each parent facility to identify psychiatrists and/or other prescribing providers to work as part of the tele-PCMHI team.

### Adaptation and refinement (specific aim 1)

#### Expert panel

Prior to adapting PCMHI, we will conduct an expert panel comprised of clinical providers and managers who are applying telemedicine to identify core components of a tele-PCMHI model for CBOCs. Next, we will create an implementation checklist for clinics to use to develop local implementation plans. This checklist will be reviewed by senior VA PCMHI leadership and revised based on their input to ensure that it addresses national VA standards.

#### Implementation planning

The CBOC primary care director at each site will be asked to identify a champion for program implementation. Local champions will likely be mental health staff who will be involved in supporting the delivery of the tele-PCMHI model or supervisors such as clinic manager. As part of initial IF activities [15], members of the facilitation team will conduct pre-site visit phone calls with the CBOC executive director, on-site mental health providers, and the site champion to discuss potential barriers to and facilitators of implementation, and how the program should be best implemented, given the site’s organizational context. In addition, we will request that each informant anonymously complete the Organizational Readiness for Change scale [16] to provide additional information to the facilitators and the site champion about potential implementation barriers and facilitators. The information collected in the pre-site visit phone calls will be reviewed and organized for each site to inform the implementation process. The research team has used these techniques successfully in the past to implement innovations in VAs [17].

#### Adaptation and refinement

The two facilitators will visit each CBOC to provide academic detailing on the tele-PCMHI model and meet with key stakeholders to help them adapt the program to meet local needs prior to implementation. Stakeholders will include on-site champions, mid-level mental health providers, nursing and primary care leadership as well as off-site telephone nurse care managers, tele-psychiatrists, and the tele-PCMHI clinician. The IF team will adapt the tele-PCMHI model for each participating CBOC while retaining the core elements identified by the expert panel.

#### Implementation

The tele-PCMHI clinicians will be trained by research team members or collaborators with expertise in PCMHI. Throughout the implementation, the IF team will continue the facilitation activities at each site. Throughout all phases, facilitators will track their activities in a time motion tracking log [17] and report on key facilitation activities and implementation status during implementation team meetings, and participate in weekly debriefing calls with a member of the evaluation team (qualitative expert and implementation scientist, KD), who will document all discussions during implementation team meetings and conduct weekly debriefings with the facilitators to document facilitation and adaptation in detail.

### Effectiveness trial (specific aim 2)

#### Overview/study design

The proposed study is a hybrid type 2 pragmatic effectiveness-implementation trial of tele-PCMHI in six sites over 24 months. Hybrid type 2 research trials include both effectiveness and implementation research objectives [14] to speed the process of gathering and translating evidence of the effectiveness of an innovation into implementation in real-world settings. Aim 2 will evaluate the effectiveness of tele-PCMHI in rural CBOCS and will also study the process and outcomes of the implementation strategy, IF.

**Fig. 1:**
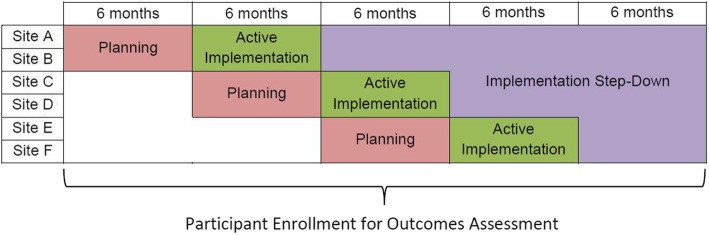
Study timeline and stepped-wedge design

The proposed study will employ a cluster-randomized, stepped-wedge design in which study sites sequentially begin implementation of the tele-PCMHI model in three waves or steps at 6-month intervals [18]. Each step will include (1) a 6-month period of implementation planning, followed by (2) a 6-month period of active implementation and (3) subsequent stepped-down implementation facilitation—that is, the research team hands off skills to continue the innovation to site personnel to enhance sustainability (see Fig. 1). The stepped-wedge design will allow us to (1) extend implementation support to the maximal number of clinics, and (2) enhance the formative evaluation of our implementation process [19]. The proposed study design also employs many core elements of pragmatic comparative effectiveness trials including (1) comparing the innovation to a commonly used active treatment, (2) applying relatively few exclusion criteria, (3) enrolling a diverse set of patients, (4) delivering the innovation using clinical staff available in routine care settings, (5) monitoring, but not controlling fidelity, (6) defining clinical outcomes as changes in patient-reported symptoms, and (7) using intent-to-treat analyses to examine group differences [20].

Patients at CBOCs that have not yet implemented the tele-PCMHI model will receive usual treatment, which in CBOCs typically consists of referral of patients with identified or suspected mental disorders to a specialty mental health care provider on-site or at a VA Medical Center farther away or through telehealth. Once the adapted tele-PCMHI has been implemented at a CBOC (Fig. 1), patients at that CBOC will have access to PCMHI services, including warm handoffs and same-day access to mental health care, in addition to the availability of referral to specialty mental health care.

Evaluation will include formative and summative evaluation activities. Formative evaluation is designed to identify potential and actual influences on the progress and effectiveness of implementation efforts and may be done before (developmental FE), during (implementation- and progress-focused FE), and/or after (interpretive FE) active implementation efforts depending upon study needs and design [19]. The formative evaluation for this project will be conducted throughout all phases of facilitation and implementation for all study sites. Summative evaluation will be conducted to quantitatively assess the effectiveness of tele-PCMHI implementation.

#### Balancing sites across implementation steps

Six CBOCs will participate in the study, with two CBOCs allocated to each of the three start dates. Because sites may differ with regard to organizational/program characteristics, we will use the restricted selection method of randomization to balance key site characteristics over time. For example, if somewhat larger CBOCs (serving a relatively more urban population) were randomized to participate in the first wave of implementation and the smallest sites were assigned to the last wave, fewer rural patients would likely be exposed to the innovation. Following the methods of Bauer and colleagues [21], we will utilize a computer-based algorithm to balance site characteristics as much as possible using key site characteristics recommended by clinical partners, including the following:

- CBOC size (number of unique patients)
- Parent facility
- Number of specialty mental health providers available on-site or via telehealth
- Measures of access to mental health care

The small number of sites and multiple categories of site characteristics rule out perfect balance. The algorithm provides as much balance as possible in the spirit of an analysis of variance (ANOVA) balanced incomplete block design.

#### Participant identification and recruitment

We will initiate recruitment for primary data collection at all sites at the same time that we begin implementation planning at the first two sites. Since the duration of the implementation period may vary slightly from site to site, we will consider a site’s first referral to the tele-PCMHI model as the index date with all patients enrolled prior to that date designated as controls and all patients enrolled after that date designated as innovation participants. Patients who screen positive on routinely administered mental health screens (i.e., depression, alcohol, PTSD) will be eligible for the study. We will exclude only those patients receiving specialty mental health treatment in the 6 months prior to recruitment; those with a diagnosis of substance dependence; and those with a psychotic disorder diagnosis (schizophrenia, bipolar disorder, and other psychotic disorders). Patients enrolled in specialty mental health care are already receiving a higher level of care than tele-PCMHI and would not be expected to benefit clinically from tele-PCMHI. The justification for excluding patients with substance dependence or psychotic disorder diagnoses is that currently there are no evidence-based PCMHI innovations for such patients.

We aim to identify and enroll patients as soon as possible after a positive mental health screen is recorded so that research assessments will capture pre-treatment severity of symptoms and functioning. Using administrative databases, we will identify all patients at study clinics who have an upcoming primary care appointment within 30 days and send them information about the study. Patients who do not opt out within 2 weeks, who have a positive screen for depression, alcohol abuse, or PTSD recorded in the administrative database, and who meet inclusion/exclusion criteria will be contacted by phone. Those who are unable to understand or engage in the informed consent process (e.g., cognitive impairment, intoxication) will be excluded. Some patients who were not identified as having an upcoming appointment may visit their clinic and have a positive mental health screen recorded. These patients will also be sent opt-out letters and contacted in 2 weeks if they do not opt out. The recruitment phase will last for 3 years.

#### Formative evaluation methods

Developmental, implementation-focused, and progress-focused formative evaluation will be conducted through detailed documentation and analysis of facilitation activities by the team’s qualitative expert and implementation scientist (author KD), who will (1) attend all weekly meetings of the implementation team, documenting all discussions and (2) conduct debriefings with the two study facilitators each week. Notes from these meetings will be cleaned, organized by site, and uploaded into Atlas.ti qualitative data analysis software for analysis. Data analysis will include a hybrid inductive-deductive approach, with deductive codes informed by the Integrated Promoting Action on Research Implementation in Health Services (i-PARIHS) framework [22] and inductive codes derived from emerging themes in the data [23, 24]. Findings will be fed back to the implementation team periodically during implementation team meetings in order to inform ongoing facilitation and implementation efforts.

We will also conduct patient interviews for our progress-focused formative evaluation [19]. We will interview four patients at each site during early implementation to assess patient perspectives on barriers to and experiences with tele-PCMHI (*N* = 24). Patients will be identified by a tele-PCMHI provider who will provide names and contact information via encrypted e-mail, telephone, or secure research website to the evaluation team. Potential patient participants will be sent an opt-out letter and recruited by phone if they do not opt-out within 2 weeks. Because we need to interview patient participants soon after they receive their mental health care, we will obtain verbal consent over the phone and document that we have obtained verbal consent in the research record. The evaluation team’s qualitative expert (author KD) will conduct these interviews using a semi-structured interview guide covering the topics of patient experience with the telehealth innovation, barriers encountered in receiving services via telehealth, and preferences for receiving care via telehealth or otherwise. These results will be fed back to the IF team to further refine how tele-PCMHI is being implemented and improved at each site.

#### Interpretive evaluation

The objective of the interpretive evaluation is to obtain stakeholder perspectives post-implementation on the perceived value of and satisfaction with the implementation process, barriers and facilitators encountered, unintended consequences, and any needed refinements to future iterations of the innovation. To obtain these perspectives, evaluation team qualitative expert (author KD) will also conduct qualitative interviews with key site personnel identified by the facilitators (e.g., clinic director, site champion) following implementation to obtain their feedback on implementation processes and facilitation efforts [19, 21]. These data will be collected primarily for the purposes of triangulating with our quantitative summative evaluation data (see below) to confirm and/or explain summative evaluation findings for each site. Finally, we will synthesize qualitative findings with quantitative findings to develop an individual descriptive case study of how facilitators helped to further adapt and implement Tele-PCMHI at each study site (see Additional file 1).

#### Summative evaluation measures

##### Overview

The hybrid type 2 effectiveness-implementation trial will be evaluated using the RE-AIM Framework [25–28]. The study timeline will not allow us to fully study the maintenance of the innovation. To have a broad public health impact, an innovation must reach a large proportion of the targeted patient population, be adopted by providers, be implemented with high fidelity, effectively improve clinical outcomes, and be maintained after the research funds are withdrawn. By measuring the innovation’s *Reach* and *Effectiveness* in the patient population of interest, we will be able to estimate the “population level impact” of PCMHI [29]. We are planning to extend traditional measurements of *implementation* by evaluating fidelity to the innovation, implementation process, and the implementation strategy using multiple measures. See Additional file 1 for our operational definitions of RE-AIM domains.

We will use methods we have employed previously to extract and analyze administrative and clinical data from the VA Corporate Data Warehouse [30], including service use data, mental health diagnoses (ICD-10), mental health screening results, age, gender, marital status, percent disability, and zip code. These data will be used to identify patients who are eligible for the effectiveness trial and to assess service use and determine RE-AIM measures at the patient- and CBOC-levels.

##### Reach

The primary measure of reach into the patient population will be the percentage of all patients with positive mental health screens that received any PCMHI services at the time of the screening visit or within the next 6 months. This measure will indicate how much the innovation is reaching the *intended* target population. A secondary measure will be a national VA performance measure of PCMHI penetration that will indicate how much the innovation is reaching the general patient population: the percentage of assigned primary care patients who receive at least one PCMHI service. Both measures will be determined using administrative data.

##### Effectiveness

We will administer research assessments to enrolled patients at the time of enrollment and at 6-month follow-up. Assessments will be administered via telephone by trained research assistants using a computer-assisted telephone interviewing system. Table 1 lists the instruments.

**Table 1:** Effectiveness assessment instruments

Instrument	Construct
Administred only at baseline^a^	
Socio-demographics	18 items that measure socio-economic and military characteristics
Hoge barriers assessment	14-item measure of perceived access, need, and treatment effectiveness [34]
Perceived access inventory^b^	43 items that perceived access to mental health care instrument developed in a preceding research project
Readiness ruler	3 items that assess perceived readiness to seek treatment [35]
Administered at baseline and follow-up	
SF-12V^c^	12 items addressing overall physical and mental health functioning [31]
Patient Health Questionnaire-9^d^	9-item inventory that yields a continuous and dichotomous assessment of depression [32]
Alcohol Use Disorders Identification Test (AUDIT-C)^d^	3 items that yield a continuous and dichotomous assessment of alcohol use [33]
Miklowitz Adherence Scale	2-item medication adherence scale [36]
Pain scale	Single-item participant rating of the average overall level of pain for the past week rated on a continuous scale from 0 to 10
Jenkins Sleep Scale	4-item measure that assesses trouble falling and staying asleep, and feeling tired during the daytime [37]
Prime-Screen	6-item assessment of dietary and exercise habits/behaviors [38]
Generalized anxiety Disorder 7-item	7-item inventory that yields a continuous and dichotomous assessment of generalized anxiety disorder [39]
American Psychiatric Association–Diagnostic and Statistical Manual of Mental Disorders (DSM) Severity Measure for Panic-Adult	10-item inventory that yields a continuous and dichotomous assessment of panic disorder [40]
PTSD Checklist for DSM-5	20-item inventory that yields a continuous and dichotomous assessment of posttraumatic stress disorder (PTSD) [41]
Behavioral Risk Factor Surveillance System Tobacco Use	5 items to assess current tobacco use
Client Satisfaction Questionnaire	8 items to assess client satisfaction with MH services [42]

^a^Constructs assessed via these instruments will be used for case-mix adjustment

^b^The Perceived Access Inventory was developed in 2017 as the main product of another VA research project [43]

^c^Measures for primary effectiveness outcome

^d^Measures for secondary effectiveness outcomes

We will assess the effectiveness of tele-PCMHI regarding improving patient outcomes for two different patient subgroups. The first patient subgroup (subgroup A) will consist of patients who screen positive for only depression or alcohol misuse in primary care—this is the subgroup for whom PCMHI is targeted based on current effectiveness data. The second patient subgroup (subgroup B) will consist of patients who screen positive for posttraumatic stress disorder (PTSD) with or without depression or alcohol misuse in primary care. The effectiveness of PCMHI has not been documented for this patient subgroup on mental health symptom outcomes and therefore, measures of PCMHI effectiveness will be explored and conceptualized differently for subgroup B compared to subgroup A.

For subgroup A patients, the primary measure of effectiveness will be clinically and statistically (*p* < 0.05) significant reductions in symptoms of depression and alcohol misuse. This primary analysis attempts to replicate current findings that traditional PCMHI delivered in person leads to significant reductions in depression [44–47] and alcohol misuse [48–50]. The secondary measure of effectiveness for subgroup A will be statistically significant reductions in other psychosocial symptoms not specific to mental health: mental health functioning, physical health functioning, pain, sleep, dietary and exercise behaviors, smoking, and medication adherence. These outcomes are indicative of a patient’s whole health and may change independently of, or in addition to, disease-specific mental health symptoms and are emphasized as important markers of patient health by the VA Office of Patient-Centered Care and the Institute for Healthcare Improvement [51].

For subgroup B patients, the primary measure of effectiveness will be the percentage of patients with at least one specialty mental health care visit after a tele-PCMHI visit. This measure is conceptualized as successful referral management—that is, a patient in need of specialty mental health for PTSD was referred to and attended one specialty mental health care visit. Referral management is one of the explicit purposes of PCMHI, although not the main purpose [4, 52]. Two other exploratory measures of effectiveness for subgroup B patients will be changes in these mental health symptoms: depression, alcohol misuse, generalized anxiety disorder, panic disorder, and PTSD; and also changes in other psychosocial symptoms not specific to mental health: mental health functioning, physical health functioning, pain, sleep, dietary and exercise behaviors, smoking, and medication adherence.

##### Adoption

The primary measure of adoption will be the percentage of primary care providers referring at least one patient to tele-PCMHI per site. This will determine how many primary care providers are “adopters.” The secondary measure of adoption will be the mean percentage of primary care providers’ patients who are referred to tele-PCMHI. This will assess the extent of adoption. We will use VA administrative data to determine each primary care physician’s patient caseloads. We will classify each patient as having received tele-PCMHI services if this was documented by stopcodes in the electronic medical record during their initial encounter or during the 6-month follow-up period.

##### Implementation

We will assess implementation fidelity in both traditional ways (i.e., fidelity to the innovation) and novel ways (i.e., fidelity to the implementation process and strategy). Regarding innovation fidelity, we will assess fidelity at the program-level by assessing whether core components of the PCMHI program are in place after implementation as well as performance of the PCMHI program on same-day access measures. Same-day access is a critical and unique component of PCMHI that provides patients with access to PCMHI the same day they also have a primary care physician appointment. We will also assess innovation fidelity at the clinical encounter or visit-level by assessing self-reported behaviors of the tele-PCMHI mental health providers to evaluate how consistent they are with ideal clinical encounter behaviors. Regarding implementation fidelity, we will assess fidelity to the process of implementing tele-PCMHI using our pre-established implementation checklist. We will also assess fidelity to the implementation strategy (i.e., implementation facilitation) using an implementation fidelity monitoring tool currently in development [53].

#### Statistical analysis

##### Overview

Analyses of reach and effectiveness of the innovation will be both descriptive and inferential, comparing dependent variables for the exposed tele-PCMHI model innovation period (innovation phase) to the unexposed observation periods (control phase). Analyses of adoption and implementation will be descriptive in nature. Characteristics of the patients and the CBOCs will be summarized by exposure status to examine potential selection bias or lack of balance [54]. Because we have a small number (3) of randomization steps, we will compare numbers of patients analyzed, cluster (CBOC) size, CBOC characteristics, and patient characteristics by randomization group.

The CBOC-level analysis will examine RE-AIM measures before and after implementation of tele-PCMHI-based care. At each facility, we will determine the index start date at which the first utilization of the telemedicine-based service began. This will allow us to consider the differing lengths of time it may take to implement the innovation at various CBOCs. Before this index date, patients participating in the study at that CBOC will be considered as being in the control phase and those participating after the index date will be considered in the innovation phase. Analyses comparing before to after implementation outcomes will be conducted for continuous measures using linear mixed models. The interaction of innovation by CBOC will test whether results differ by CBOC. We will adjust for baseline differences among CBOCs in the model [55].

The patient-level analysis will be a generalized linear mixed model which includes fixed terms for innovation (usual care referral model versus tele-PCMHI care) and time (6-month time periods for each step). The model will use random effects to model the correlation of patients within CBOCs [56, 57]. In addition, we may consider adjusting the analyses to deal with lags in the innovation effect. This delayed innovation effect occurs if the innovation does not become fully effective during the step in which it is introduced [58]. If, for example, we expect the lag in the innovation effect to be 50% in the implementation step and 100% effective within 6 months, we could follow the suggestion of Hussey and Hughes of using fractional values for the treatment indicator [56].

Because this is a cross-sectional design in which different patients are sampled at each step in the study, we do not need to include a random-effects term for repeated measures on the same individuals. The effect size adjusted for calendar time and its 95% confidence interval will be calculated to assess the estimated change in outcomes after the introduction of the innovation.

Per Hemming et al. [54], we will report the estimated intra-cluster correlation for use in the design of future trials. In addition, we will report the time effect from the fitted model to allow assessment of possible confounding effects of calendar time. We may include an effect modifier term in the model representing the length of the period up to the current observation during which the CBOC has been exposed to the innovation. This will allow examination of the way the impact of the tele-PCMHI-based care develops over time once introduced into the CBOC.

##### Data analysis for reach

The first type of analysis for reach will be descriptive. We will compare the primary measure of PCMHI reach before implementation to active implementation. We will also compare the secondary measure of PCMHI penetration to the established VA performance expectation that 7% of assigned primary care patients receive at least one PCMHI service.

The second analysis for reach will be correlational. A dummy variable representing implementation group assignment (pre-implementation control or active implementation) will be specified as the explanatory variable of interest for reach. We hypothesize that during active implementation, the reach of PCMHI will be greater than during the pre-implementation period. An alpha significance level of 0.05 will be used to reject/accept the null hypothesis. Significant intra-class correlation violates the independence assumption of standard regression models and may cause underestimation of coefficient standard errors, possibly leading to incorrect inferences concerning the rejection of the null hypotheses. Therefore, the first step of the statistical analysis will be to test for lack of independence among observations within clusters using intra-class correlation coefficients at the site level. Specifically, using a likelihood ratio test, we will compare the −2log likelihoods for an unrestricted model to a model that restricts the intra-class correlation to be zero. Raudenbush recommends that unconditional models (i.e., without explanatory variables) should be estimated prior to considering conditional models (i.e., with explanatory variables) [59]. If it turns out that the −2log likelihoods are not significantly different, the hypotheses will be tested using a standard logistic regression model. The following case-mix factors will be included in the regression equation: mental health diagnostic categories, age, gender, race, marital status, percent disability, and rurality. Missing race data will be handled by specifying an unknown race category. Conversely, if the results of the likelihood ratio test suggest that the intra-class correlation is significant, a mixed logistic model will be used [60, 61]. The mixed-model will include a random effect for the intercept and fixed effects for the patient-level variables (including implementation group assignment). The variance-covariance matrix will be specified to be unstructured.

##### Data analysis for effectiveness

For patients screening positive for depression or alcohol use only (subgroup A), we will calculate change scores between baseline and 6-month follow-up for (a) depression and alcohol misuse and (b) whole health outcomes (i.e., mental health functioning, physical health functioning, pain, sleep, dietary and exercise behaviors, smoking, and medication adherence). The primary outcome of interest for the effectiveness analysis is change in overall mental health functioning (Short Form 12–Veterans Version [SF-12 V] Mental Health Composite Scale mental health composite score) [31]; secondary effectiveness outcomes are changes in depressive symptoms (PHQ-9) [32] and alcohol use (AUDIT-C) [33]. See Table 1 for assessments. For patients screening positive for PTSD (subgroup B), we will examine the extent of referral management, defined as the proportion of patients who have a specialty mental health encounter following a tele-PCMHI encounter, and calculate change scores between baseline and 6-month follow-up for mental health symptom measures and whole health measures. Hierarchical models will be run in which patients are nested within the CBOC in which they received care. We will adjust for covariates selected a priori to prevent overfitting [61]. We hypothesize there will be a significant effect of tele-PCMHI on the differences between baseline and 6-month follow-up scores on all the aforementioned effectiveness measures. An alpha significance level of 0.05 will be used for all analyses.

##### Data analysis for adoption

Descriptive analysis will determine the proportion of primary care providers who refer at least one patient to tele-PCMHI after implementation, and the mean number of patients referred per provider.

##### Data analysis for implementation fidelity

Analyses will be descriptive. Implementation fidelity at the program (CBOC) level and provider level [62] and fidelity to the implementation strategy will be determined as described in Additional file 1.

##### Power calculation

We calculated the sample size needed given the stepped-wedge design to test the primary hypothesis, that the innovation improves the primary outcome, SF-12 V Mental Health Composite Scale scores, following the approach of Woertman et al. [63], which has been corrected by Hemming, Girling, and Taljaard [64, 65]. The calculations are based on having 80% power at a significance level of 0.05 to detect a medium effect size (Cohen’s *d* = 0.50) between the usual care referral model and the tele-PCMHI model innovation group in our primary outcome measure.

The total number of patients to be selected for assessment across the 6 CBOCs will be 540. This was determined after adjusting the total sample size required under individual randomization *N*_u_, for the design effect, multiplying by the 5 measurement periods, and accounting for a 20% attrition rate. This will result in approximately 432 patients providing baseline and 6-month follow-up data for analysis. Thus, we aim to recruit 18 patients in each of the 5 6-month periods at each of the 6 CBOCs.

## Discussion

This project provides a unique opportunity to study the implementation of PCMHI in rural clinics. The novel delivery of these services through telehealth technologies mitigates barriers frequently experienced by both providers and veterans served in these settings.

First, the ability to hire specialty mental health providers in rural community-based settings is a long-standing challenge both in and outside of the VA. To address this barrier, the VA has traditionally provided a referral to part-time specialty mental health services or specialty mental health care provided by scheduled appointments. Yet, these services do not address the needs of the population targeted by PCMHI, veterans with mild to moderate symptoms which, if addressed when they came to an appointment in primary care, would not require specialty mental health care. Moreover, a referral usually necessitates an additional visit to the clinic or to a distant VA medical center, adding additional barriers. In addition, the volume of veteran patients with mental health needs in some rural clinics may not be enough to justify on-site PCMHI teams. Finally, as currently configured, mental health care for those living in rural settings is often not equitable to what is available in larger or medical center clinics. The VA is committed to providing veterans “the right care, in the right place, at the right time [66].” The novel application of tele-PCMHI described here will provide the same open access, integrated services that PCMHI and similar models provide in other settings [3, 5–8].

The primary limitation of the study is that it is taking place at a time of rapid change in VA mental health care, with ongoing implementation of PCMHI, including some instances of tele-PCMHI. Formative evaluation will carefully document any such changes at the study sites.

## Additional file

Additional file 1: RE-AIM framework evaluation plans. (DOCX 35 kb)

## Abbreviations

ANOVA: Analysis of variance
CBOCs: Community-based outpatient clinics
IF: Implementation facilitation
i-PARIHS: Integrated Promoting Action on Research Implementation in Health Services
LCSW: Licensed Clinical Social Worker
PCMHI: Primary Care Mental Health Integration
PDSA: Plan-Do-Study-Act
PPAQ: Primary Care Behavioral Health Provider Adherence Questionnaire
PTSD: Posttraumatic stress disorder
RE-AIM: Reach Effectiveness Adoption Implementation Maintenance
Tele-PCMHI: Telehealth model of Primary Care Mental Health Integration
VA: Veterans Health Administration
VISN: Veterans Integrated Service Network
