# Supplementary material for: Using implementation facilitation to implement primary care mental health integration via clinical video telehealth in rural clinics: protocol for a hybrid type 2 cluster randomized stepped-wedge design
Source: Implement Sci. 2019 Mar 21;14:33. doi: 10.1186/s13012-019-0875-5 (PMC6429823; doi:10.1186/s13012-019-0875-5)
Supplement: Supplementary file 1 — RE-AIM framework evaluation plans. (DOCX 35 kb) [file 13012_2019_875_MOESM1_ESM.docx]

| **Construct** | **Original Definition [25]** | **Operational Definition** | **Data Collected** |
| --- | --- | --- | --- |
| Reach | Target population exposed to innovation | Proportion of patients receiving tele-PCMHI, including:  Subgroup A: % of primary care patients with mental health problems who have contact with tele-PCMHI  Subgroup B: % of primary care patients seen in tele-PCMHI | Subgroup A (within site %):  Patients with positive mental health screens in primary care and 1+ tele-PCMHI visit  _____________________________________  Patients with positive mental health screens in primary care  Subgroup B (within site %):  # patients with 1+ tele-PCMHI visit  _____________________________________  # total patients in primary care |
| Effectiveness | Success rate and clinical impact if innovation implemented with fidelity | Patient outcomes, including:  Subgroup A: Patients who screen positive in primary care clinic for depression or alcohol abuse only  Subgroup B (exploratory): Patients who screen positive in primary care clinic for PTSD with or without depressive symptoms and/or alcohol misuse | Subgroup A:^[[1]](#footnote-2)^   - 1. Significant reduction in depressive symptoms and alcohol misuse after implementation of tele-PCMHI.   2. Significant reduction in other psychosocial domains indicative of patient’s whole health (i.e., medication adherence, functioning, pain, sleep, dietary and exercise behaviors, tobacco use).   Subgroup B:^[[2]](#footnote-3)^   1. Percentage of patients with at least one specialty mental health care visit 2. Significant reduction in these mental health symptoms after implementation of tele-PCMHI: depression, alcohol misuse, generalized anxiety disorder, panic disorder, and PTSD 3. Significant reduction in other psychosocial domains indicative of patient’s whole health (i.e., medication adherence, functioning, pain, sleep, dietary and exercise behaviors, tobacco use). |
|  |  |  |  |
| Adoption | Populations adopting the innovation | 1. % of Primary care providers referring at least one patient to tele-PCMHI 2. Proportion of Primary care providers’ patients referred to tele-PCMHI | 1. # of Primary care providers with 1+ referral to tele-PCMHI   _____________________________________  total # of Primary care providers   1. # of patient referrals to tele-PCMHI per PCP   _____________________________________  PCP’s total # of patients in panel |
| Implementation | Innovation fidelity in the real-world | 1. Innovation Fidelity 2. Fidelity at program component level    1. Program model    2. Program performance 3. Fidelity at clinical encounter (provider) level 4. Implementation Fidelity    1. Fidelity to tele-PCMHI implementation process    2. Fidelity to facilitation (implementation strategy) | 1. Innovation Fidelity   a1. Case summaries rated by experts^[[3]](#footnote-4)^  a2. Same day access = primary care encounters / tele-PCMHI encounters^[[4]](#footnote-5)^  b. Primary Care Behavioral Health Provider Adherence Questionnaire (PPAQ) [62]   1. Implementation Fidelity    1. Pre-post comparison of implementation checklist^[[5]](#footnote-6)^    2. Implementation Facilitation Fidelity Tool [53]^[[6]](#footnote-7)^ |

1. We will compare patient-level change between Time 1 and Time 2. Time 1: Before implementation, patients with positive depression or alcohol screens in primary care; Time 2: Six months after implementation of tele-PCMHI, same patients [↑](#footnote-ref-2)
2. Currently, PCMHI has effectiveness data for treating depression and alcohol misuse. This exploratory analysis will assess whether patients with psychiatric conditions other than depression or alcohol abuse (e.g., PTSD) benefit from tele-PCMHI, especially regarding successful referral management from primary care to specialty mental health. We will compare patient-level change between Time 1 and Time 2. Time 1: Before implementation, patients with a positive PTSD screen in primary care (with or without positive depression or alcohol misuse screens); Time 2: Six months after implementation of tele-PCMHI, same patients. [↑](#footnote-ref-3)
3. One case summary from each site will detail existing program components and will be compiled using information from qualitative interviews with primary care staff and leadership after implementation. Then, each case summary will be de-identified and rated by the expert panel that determined core program components for tele-PCMHI early in the implementation process. [↑](#footnote-ref-4)
4. The percentage of patients in primary care that are also seen in PCMHI that same day is currently used as a national performance measure in VA. [↑](#footnote-ref-5)
5. The implementation facilitation team will compare the implementation checklist before implementation to the implementation checklist at the end of active implementation (i.e., six months after implementation has started). [↑](#footnote-ref-6)
6. This measure is being developed by investigators at the VA QUERI Program for Team-Based Behavioral Health [53]. [↑](#footnote-ref-7)
